# Tau aggregation induces cell death in iPSC-derived neurons

**DOI:** 10.1016/j.nbas.2025.100136

**Published:** 2025-04-11

**Authors:** Hirokazu Tanabe, Sumihiro Maeda, Etsuko Sano, Norio Sakai, Setsu Endoh-Yamagami, Hideyuki Okano

**Affiliations:** a FUJIFILM Corporation, Bio Science & Engineering Laboratories, Kanagawa, Japan; b Department of Physiology, Keio University School of Medicine, Tokyo, Japan; c Keio University Regenerative Medicine Research Center, Kanagawa, Japan; d Department of Molecular and Pharmacological Neuroscience, Graduate School of Biomedical & Health Sciences Hiroshima University, Hiroshima, Japan

**Keywords:** Tau, iPSC, Neuronal cell death, Aggregation, Overexpression

## Abstract

Abnormal accumulation of tau proteins in the brain is a hallmark of neurodegenerative diseases such as Alzheimer’s disease and is closely linked with neuronal cell death. Tau accumulation is a prominent therapeutic target for Alzheimer’s disease, since tau accumulation correlates well with the disease progression, and tau-targeting drugs hold potentials to halt the disease progression. Given the differential response of human and mouse neuronal cells, there is a critical need for a human cellular platform to quickly screen for tau-related neurodegenerative disease therapeutics. However, inducing rapid, tau-dependent neuronal cell death in human models remains challenging. In this study, we established a human cellular model capable of inducing tau aggregation-dependent neuronal cell death within two weeks via tau overexpression. Additionally, we demonstrated the neuroprotective efficacy of known tau-targeting compounds within this system. These findings suggest that our cellular model recapitulates the molecular pathogenesis of tau-induced neurodegeneration and could serve as a valuable platform for drug screening in tauopathies.

## 1 Introduction

Tau is a microtubule-associated protein that is abundant in neurons, and the primary physiological function of tau is to regulate microtubule stability [1,2]. However, under pathological conditions, tau proteins form filamentous polymers and accumulate in patient’s brains, which are called neurofibrillary tangles (NFTs) [3,4,5]. NFT is a histopathological hallmark of various neurodegenerative diseases such as Alzheimer’s disease (AD), frontotemporal lobar degeneration (FTLD), and progressive supranuclear palsy (PSP) [3,4,5]. NFTs exhibit a stereotypical spatiotemporal progression that correlates with the severity of cognitive decline in AD [5,6,7]. The progression is also associated with cognitive impairment due to executive dysfunction in PSP [8]. Accumulation of NFTs is linked to neuronal loss, suggesting that the progression of NFT formation is involved in brain dysfunction [9,10]. Tau protein aggregates into oligomers, and further aggregation leads to filamentous polymers. In particular, tau oligomers have attracted significant attention in tau-related diseases due to their association with cytotoxicity and their increase in the early stages of the pathogenesis [11,12,13]. However, the mechanisms underlying their toxicity and the contribution of different forms of tau aggregates including tau oligomers remain unclear and require further study.

Under normal physiological conditions, there is a dynamic balance between tau binding to and unbinding from microtubules, which is primarily regulated by the phosphorylation status of tau [3,14]. Direct and indirect pathological events lead to aberrant tau phosphorylation, detachment of tau proteins from microtubules, and formation of tau aggregates [3,14]. It is increasingly accepted that neurotoxicity is more likely due to toxic gain of function rather than loss of function, as *tau* knockout mice do not show neuronal cell loss, while tau over-expression induces neuronal cell loss [15]. The formation of protein aggregates, followed by their acquisition of cytotoxicity, is a widespread phenomenon in neurodegenerative diseases, and various approaches have been taken to analyze the mechanisms of toxicity [16,17,18]. Neurotoxicity due to tau perturbation has been mainly studied using tau transgenic mouse models expressing human tau proteins [3]. However, it is noted that the susceptibility of neurons to toxic tau aggregates differs between human and mouse neuronal cells [19]. Hence, the use of human induced pluripotent stem cell (iPSC)-derived neurons is expected to provide a better understanding of tau’s functions in human cells and offer insights into the study of neurodegenerative diseases [20,21]. On the other hand, it is difficult to establish iPSC-derived neuronal models of age-related neurodegenerative diseases because iPSC-derived neurons have the characteristics similar to fetal neurons due to rejuvenation during the reprogramming process to generate iPSCs [21,22]. Although tau aggregation and neuronal cell death have been demonstrated in iPSC-derived neuronal models, the requirement for a long culture period makes it difficult to use these models as a tool for drug discovery research. On the other hand, exogenous tau overexpression has been used as a method to increase intracellular aggregated tau in immortalized cell models [23]. However, it is challenging to demonstrate both tau aggregation and cell death. For instance, previous studies have reported that the combination of tau overexpression and aggregation inducer eventually leads to tau aggregation and cell death [24,25]. Therefore, we attempted to reproduce tau aggregation and tau-dependent cell death by tau overexpression in human iPSC-derived neurons within a short period of time. This system utilizes rapidly induced neurons that were generated by the expression of Ngn2 and miR-9/9*-124, along with lentiviral vectors integrating the TET-on system, which enables efficient tau expression within a short timeframe. Most cellular assays for tau toxicity have relied on tau mutants; however, in tauopathies such as AD, wild-type tau, rather than mutant tau, accumulates. Therefore, we aimed to develop a cellular assay that accurately models the toxicity of wild-type tau. Using this assay, we evaluated several compounds in a neuronal model, demonstrating its potential as a drug discovery platform for tau-related neurodegenerative diseases. Establishing a system that replicates key pathological features of neurodegenerative diseases, tau aggregation and cell death, without requiring long-term, complex cell culture offers a significant advantage for the development of novel therapeutic drugs.

## 2 Material and methods

### 2.1 Preparation of lentivirus for tau overexpression and miR-9/9*-124 expression

Self-inactivating (SIN) vector for lentivirus was constructed by the method using a Multisite Gateway-based method as previously described [26]. SIN vectors for overexpression (CSII-TRE-1N4R-IRES-zeo, CSII-TRE-1N4R (A152T)-IRES-zeo, CSII-TRE-1N4R (P301S)-IRES-zeo, CSII-TRE-0N3R-IRES-zeo, CSII-TRE-TagGFP2-IRES-zeo, CSII-TRE-IRES-zeo, and CSII-TRE-1N4R (ΔPHF6)-IRES-zeo) were constructed by the partially modifying method as previously described [26]. First, primers were designed to contain the attL1 and attL2 sequences at both ends of the overexpressing gene sequence, and PCR was performed using the overexpressing gene as the template to prepare the PCR product with attl1 and attL2 at both ends. After that, the vectors and CSII-EF-MCS vector were genetically recombined using the Gateway method [27,28]. Using these SIN lentiviral vectors (LV), each lentiviral particle was prepared by the method previously described [29].

### 2.2 Establishment of iPS cells and feeder-free culture

FF-1 human iPSC line (provided from Fujifilm Cellular Dynamics, Inc., WI, USA) was used in this study. We edited the *MAPT* gene of FF-1 iPSC line to fuse TagGFP2 to the N-terminus of the *MAPT* gene in heterozygote by gene editing as previously described [26] and named FF-1_N-GFP-tau cell line.

The feeder-free iPSC culture was prepared as previously described [26,29]. Briefly, iPSCs were maintained in StemFit/AK02N, and passaged every 6–8 days. iPSCs were cultured onto a pre-coated 6-well plate with iMatrix-511 silk. On the first day, iPSCs were cultured in the presence of 10 µM Y27632, then the culture medium was exchanged to the fresh medium every 1 or 2 days.

### 2.3 Establishment of Ngn2 + miR-9/9*-124 introduced iPSC line and preparation of iPSC-derived neurons

FF-1_N-GFP-tau cell line was transfected with pCMV-HyPBase-PGK-Puro (CMV-HyPBase-PGK-Puro), PB-TET-PH-lox66FRT-NEUROG2 (ITR-PGK-Puro-TRE-Ngn2-IRES-βgeo-ITR), PB-CAGrtTA3G-IH (ITR-CAG-rtTA-IRES-Hygro-ITR) vectors, and lentivirus containing CSIV-miR-9/9*-124-mRFP1-TRE-EF-BsdT vector as previously described [26]. Stably expressing cells were selected by the selection drugs, hygromycin, puromycin, and blasticidin S. Single cell-derived clones were isolated from the survived clones after the drug selection. The clones were further selected based on the capability of neuronal differentiation by expressing Ngn2 and miR-9/9*-124. These iPSC lines were maintained slightly modified method from the above feeder-free cell culture method. Briefly, on day 3–5 after seeding, drug selection was performed by adding hygromicin (50 µg/mL), puromycin (0.5 µg/mL), and blasticidin S (10 µg/mL) to StemFit.

The selected clones of iPSCs were differentiated to neurons after driving the expression Ngn2 and miR-9/9*-124 by DOX treatment with the Neuronal induction medium, which is composed of Neurobasal Plus medium containing 2 % B27 Plus supplement, 1 % CultureOne supplement, 1 % GlutaMAX, 200 µM L-ascorbic acid, 200 µM dbcAMP, 10 µM Y27632, 20 µM DAPT and 4 µg/ml DOX, for 5 days. On day 0, iPSCs were seeded onto culture plates pre-coated with 75 µg/mL Poly-L-ornithine and 5 µL/mL iMatrix-511 silk. On day 2, half medium change was performed with the fresh Neuronal induction medium described above. The prepared neurons were stocked as frozen cells using STEM-CELL BANKER, or seeded to plates immediately.

### 2.4 Tau overexpression

Commercially available PDL pre-coated 96 well plates were additionally coated by 5 µL/mL iMatrix-511 silk diluted with PBS and incubated overnight at 37 °C. The iMatrix-511 silk solution was removed, and 40 μL Neuronal re-seeding medium (Neurobasal Plus medium containing 2 % B27 Plus supplement, 1 % CultureOne Supplement, 1 % GlutaMAX, 200 µM L-ascorbic acid, 200 µM dbcAMP, and 2 µg/ml Dox) was added to the coated plate. 10 µL of Accell human MAPT siRNA (si-tau), Accell Non-targeting Control siRNA (si-control), isoproterenol, or epothilone D solution dissolved by Neuronal re-seeding medium at 10 times of the aimed concentrations were added to the plate. The frozen stock of neurons was resuspended by Neuronal re-seeding medium and plated at 3 × 10^4^ cells/well with 50 µL of volume (total 100 µL/well). Two days after re-plating, 10 µL of lentiviral solution (MOI (multiplicity of infection) 0.1–10) dissolved with Neuronal re-seeding medium was added to neurons for tau overexpression. Each lentivirus has *MAPT* or *TagGFP2* gene under the TET-on promoter to drive the expression after doxycycline treatment. We did not additionally introduce rtTA to the cells because the iPSC-derived neurons originally possess the rtTA transgene to drive the expression of Ngn2 and miR9/9*-124. Cell viability assay and Immunofluorescence described below were performed 5–7 days after tau overexpression.

### 2.5 Cell viability assay

To examine the neuronal viability, we measured the cell viability by CellTiter-Glo Luminescent Cell Viability Assay following the manufacturer’s protocol. Briefly, we first prepared CellTiter-Glo solution by diluting the CellTiter-Glo substrate with CellTiter-Glo buffer. Equal amounts of CellTiter-Glo solution (100 µL) were added to the wells and mixed by pipetting to induce cell lysis. After 10 min of incubation at room temperature, luminescence signals were measured with a luminescent microplate reader.

### 2.6 Immunofluorescence

Neurons were fixed by exposing to 4 % paraformaldehyde for 20 min. After washing with PBS, the neurons were blocked with blocking buffer (5 % FBS, 0.3 % Triton-X100 in PBS) for 30 min. The neurons were incubated with primary antibodies (βIII tubulin, MAP2, 4R tau, AT8, PHF1, MC1, and T22) diluted to 1/1,000 with blocking buffer overnight at 4 °C. After washing with PBS, the neurons were incubated with secondary antibodies (Goat anti-Mouse IgG (H + L) Secondary Antibody, Alexa Fluor 555, Goat anti-Rabbit IgG (H + L) Secondary Antibody, Alexa Fluor 647) diluted to 1/2,000 with blocking buffer, and 10 µg/mL Hoechst 33,342 for 1 h at room temperature. After washing with PBS, fluorescence signals (excitation 545 nm/emission 605 nm, excitation 620 nm/emission 700 nm, or excitation 360 nm/emission 460 nm) were observed with a fluorescence microscope.

### 2.7 Non-reducing SDS PAGE

Commercially available PDL pre-coated 12 well plates were additionally coated by 5 µL/mL iMatrix-511 silk diluted with PBS and incubated overnight at 37 °C. The iMatrix-511 silk solution was removed, and thawed neurons were plated at 5 × 10^5^ cells/well with 750 µL Neuronal re-seeding medium. Two days after re-plating, MOI 3 lentivirus was added to neurons for tau overexpression. Five days after lentivirus treatment, cells were lysed with mRIPA buffer (50 mM Tris-HCl (pH 7.4), 150 mM NaCl, 1 % NP-40, 0.25 % Na-deoxycholate (w/v), 1 mM EDTA). The cell lysate was boiled with NuPAGE LDS sample buffer (4 × ) to make cell extract samples. Cell extract samples, Tau protein ladder, and protein molecular weight marker were loaded to NuPAGE 4–12 % Bis-Tris gel, and performed electrophoresis (200 V, 30 min) in MOPS Running buffer. The protein on the gel was transferred to immobilon-FL PVDF membrane using NuPAGE Transfer buffer (100 mA, 60 min). After that, membranes were blocked with Odyssey Blocking Buffer (TBS) at room temperature for 30 min. Primary antibodies (Rabbit anti-Tau or Rabbit anti-beta III tubulin) diluted to 1/2,000 with blocking buffer were incubated at 4 °C for two overnight. After washing with 1 × TBST, membranes were incubated with 1/10,000-diluted secondary antibodies (IRDye 680RD Donkey anti-Rabbit IgG Secondary Antibody, IRDye 800CW Donkey anti-Rabbit IgG Secondary Antibody) by shaking at room temperature for 1 h. After washing with TBST, total tau and βIII tubulin signals were detected with the fluorescence image analyzer.

### 2.8 Statistical analysis

The statistical tests used for each dataset are specified in the figure legends. Statistical analyses were done with Prism 5.04, 7, 9 or 10. Normality was assessed with the Shapiro–Wilk normality test. Variances were assessed with the F-test (two groups) or Bartlett’s test (more than two groups). For multiple group comparisons with normal distribution, we used one-way ANOVA with post hoc Tukey or Dunnett’s test. Interaction between groups was analyzed by analysis of covariance using JMP (ver 15.0). Values reported are means ± SD. Differences were considered significant at P ≤ 0.05.

### 2.9 Reagents

The reagents and resources used in this study are listed in Table 1.

**Table 1:** 

REAGENT or RESOURCE	SOURCE	INDENTIFER
Antibodies		
Mouse anti-4R Tau, clone 1E1/A6	Sigma-Aldrich	05–804
Rabbit anti-4R Tau	Cosmo Bio	CAC-TIP-4RT-P01
Rabbit anti-Tau (K9JA)	Agilent / DAKO	A0024
Mouse anti-Phospho-Tau (Ser202, Thr205) (AT8)	Thermo Fisher	MN1020
Mouse anti-phospho Tau (PHF1)	provided by Dr. Peter Davies, Albert Einstein University	N/A
Mouse anti-oligomer Tau (MC1)	provided by Dr. Peter Davies, Albert Einstein University	N/A
Rabbit anti-Tau (T22), oligomeric Antibody	Sigma-Aldrich	ABN454
Mouse anti-MAP2	Sigma-Aldrich	M4403
Rabbit anti-beta III tubulin	Abcam	ab52623
Goat anti-Mouse IgG (H + L)Secondary Antibody, Alexa Fluor 555	Thermo Fisher	A21424
Goat anti-Rabbit IgG (H + L) Secondary Antibody, Alexa Fluor 647	Thermo Fisher	A21245
IRDye 680RD Donkey anti-Rabbit IgG Secondary Antibody	Li-cor	925–68073
IRDye 800CW Donkey anti-Rabbit IgG Secondary Antibody	Li-cor	926–32213
Hoechst 33,342	Thermo Fisher	H3570
Chemicals, Peptides, and Recombinant Proteins		
StemFit, AK02N	Ajinomoto	AK02N
iMatrix-511 silk	Matrixome	892,021
TrypLE Select Enzyme (1X), no phenol red	Thermo Fisher	12,563,029
Hygromycin B Solution	Nacalai Tesque	09287–84
Puromycin (solution)	Nacalai Tesque	ant-pr-1
Blasticidin S	Funakoshi	KK-400
Poly-L-ornithine hydrobromide	Sigma-Aldrich	3655
Neurobasal Plus Medium	Thermo Fisher	A3582901
GlutaMAX Supplement	Thermo Fisher	35,050,061
B-27 Plus Supplement (50 × )	Thermo Fisher	A3582801
CultureOne supplement	Thermo Fisher	A3320201
Doxycycline Hyclate (DOX)	Tokyo Chemical Industry	D4116
Y-27632	Nacalai Tesque	8945–42
Dibutyryl cAMP Sodium Salt (dbcAMP)	Nacalai Tesque	11540–61
L-Ascorbic acid	Sigma-Aldrich	A4544
DAPT	Sigma-Aldrich	D5942
Polyoxyethylene(10) Octylphenyl Ether (Triton X-100)	Fujifilm Wako Pure Chemical	168–11805
Paraformaldehyde	Muto Pure Chemicals	17,711
Fetal Bovine Serum (FBS)	Sigma-Aldrich	172012-500ML
Odyssey Blocking Buffer (TBS)	LI-COR	927–50000
0.05 %-tTBS(10x)(pH 7.4) (TBST)	Nacalai Tesque	12749–21
Tau protein ladder	rPeptide	T-1007–2
NuPAGE 4–12 % Bis-Tris Protein Gels, 1.0 mm, 17-well	Thermo Fisher	NP0329BOX
NuPAGE LDS sample buffer (4x)	Thermo Fisher	NP0007
MOPS Running buffer 20x	Thermo Fisher	NP0001
NuPAGE Transfer buffer 20x	Thermo Fisher	NP0006
Immobilon-FL PVDF	Merck Millipore	IPFL00010
BioCoat Poly-D-Lysine 12-well plate (PDL pre-coated 12 well plate)	CORNING	356,470
CELLCOAT, PDL, μClear, 96 well (PDL pre-coated 96 well plate)	Greiner	655,946
Accell non-targeting siRNA	Dharmacon	D-001910–03-50
Accell Tau siRNA	Dharmacon	A-012488–13-0050
5 × siRNA buffer	Dharmacon	B-002000-UB-100
STEM-CELL BANKER	Takara Bio	CB045
Critical Commercial Assays		
CellTiter-Glo Luminescent Cell Viability Assay	Promega	G7570
Recombinant DNA		
CSII-EF-MCS	RIKEN BRC	RDB04378
Software and Algorithms		
Odyssey CLx	Li-cor	−
Prism 5.04, 7, 9, and 10	GraphPad Software	−
JMP 15.0	JMP	−
All-in-One Fluorescence Microscope (BZ-800)	Keyence	−
CENTRO LB960	Berthold Technologies	−

## 3 Results

### 3.1 Tau overexpression induces neuronal cell death in iPSC-derived neurons

**Fig. 1:**
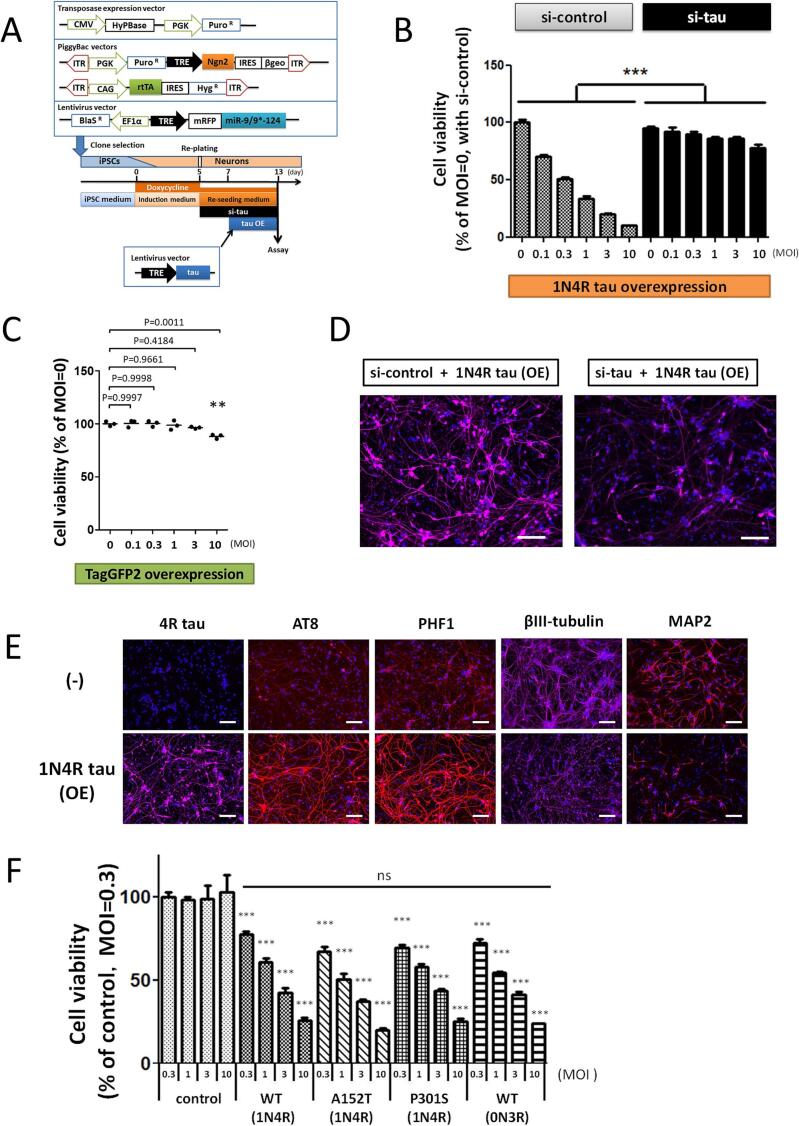
**Tau overexpression induces cell death in iPSC-derived neurons.** (A) Schematic diagram of neuronal induction from iPSCs by Ngn2 + miR-9/9*-124 expression. First, we introduced the transgenes for neuronal induction using the PiggyBac vector system into an iPSC line derived from healthy control and selected stable cell lines after drug selection. The selected iPS lines were differentiated into neurons by exposure to DOX for 5 days. On day 5, neurons were harvested and stored. The stocked neurons were thawed and plated on 96-well plates. The anti-tau or scramble control siRNAs at 1 µM (si-tau or si-control) were added upon re-plating, and lentivirus was treated with neurons for tau overexpression 2 days after re-plating. (B) Cell viability of iPSC-derived neurons at 6 days after 1N4R tau overexpression with si-control (shaded bars) or si-tau treatment (black bars) (n = 3; mean + SD). ***: P ≤ 0.001 by analysis of covariance, si-control vs. si-tau. (C) Cell viability of iPSC-derived neurons at 6 days after overexpression of TagGFP2 without tau sequence in the introduced transgene. Black horizontal lines: mean of n = 3. **: P ≤ 0.01 by Dunnett’s test, MOI 0 vs. each group. (D) Confirmation of the suppression of exogenous tau expression by the si-tau treatment. Immunofluorescence analysis using 4R tau antibody (purple) and Hoechst 33,342 (blue). Neurons were treated with 1N4R tau lentivirus (MOI 3) and fixed at 6 days after the lentivirus infection. Scale bars, 100 µm. (E) Immunofluorescence analysis using 4R tau, AT8, PHF1, βIII tubulin, MAP2 antibodies (red or purple), and Hoechst 33,342 (blue). Neurons were treated with 1N4R tau lentivirus (MOI 3) and fixed at 6 days after the lentivirus infection. (−): lentivirus-untreated group. Scale bars, 100 µm. (F) Cell viability of iPSC-derived neurons at 5 days after WT (1N4R), A152T (1N4R), P301S (1N4R), and WT (0N3R) tau overexpression was measured by CellTiter-Glo assay. Lentivirus without carrying tau was used as a negative control (n = 4, mean + SD). ns (not significant): P > 0.05 by analysis of covariance. ***: P ≤ 0.001 by Dunnett’s test, control vs. each group at the same MOI.

In order to examine whether tau overexpression induces cell death in iPSC-derived neurons, neurons were treated with lentivirus carrying a construct to induce tau expression under the TET-on promoter system, and the neurons were treated with doxycycline to overexpress tau according to the schedule shown in Fig. 1A. In the adult human brain, there are primarily six isoforms of tau proteins produced by alternative splicing: 0N3R, 1N3R, 2N3R, and 0N4R, 1N4R, 2N4R [30,31]. For this study, we selected the 1N4R isoform because 1N3R/1N4R isoforms are the most abundant isoform in adult human brains, and 4R tau is suggested to be more toxic to neurons than 3R tau. Cell viability was significantly decreased in a lentivirus MOI-dependent manner (Fig. 1B). In addition, the decrease in cell viability progressed from 4 to 6 days after tau overexpression. At post-infection day (PID) 4, only 20 % of signal reduction was found in the hTau LV infected neurons compared the non-infected neurons, while 80 % of signal was reduced at PID6 (Supplementary Fig. S1). Furthermore, tau overexpression decreased the number of cells (Supplementary Fig. S2). These results indicate that tau overexpression causes cell death overtime. Furthermore, the labeling of endogenous tau with GFP enabled us to observe the effects of exogenous tau expression on endogenous tau and neurites. Damages in the neuronal cell body and neurites were also recognized by phase-contrast images in the neurons with tau overexpression (Supplementary Fig. S3A, C), accompanied by reduction of endogenous GFP-tau fluorescence signal (Supplementary Fig. S3E, G). These data support our conclusion that cell death is induced by tau overexpression. To verify that the decreased cell viability is not simply due to protein overexpression, a lentivirus vector encoding green fluorescent protein TagGFP2 but not tau was introduced into iPSC-derived neurons. We also found a slight cytotoxic effect of GFP at MOI 10 [32], however, the effect of hTau expression on cell viability was significantly severer than that of GFP (Fig. 1C).

**Fig. 2:**
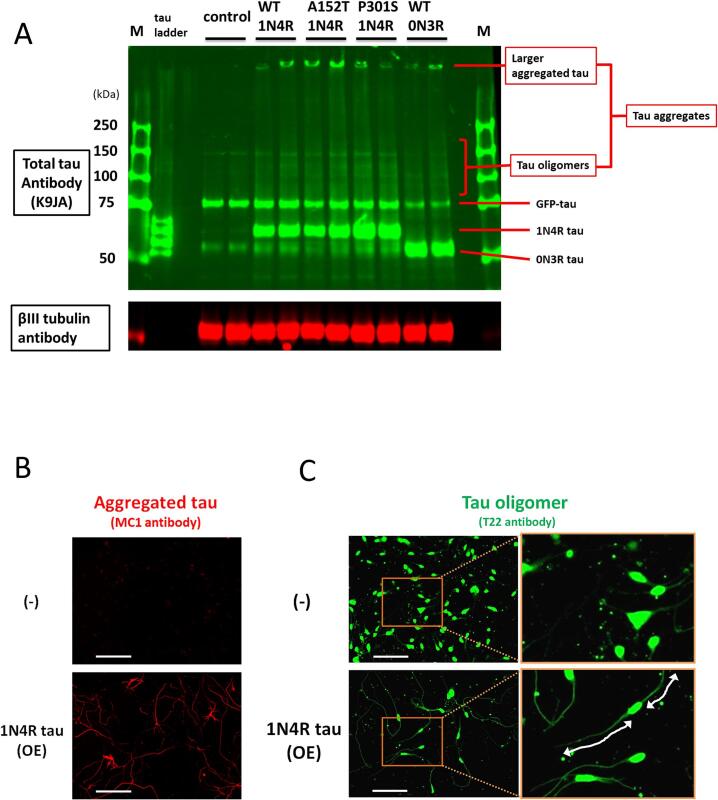
**Tau overexpression induces tau aggregation in iPSC-derived neurons.** (A) In addition to monomeric tau species, larger aggregated tau at the top of the gel and tau oligomers in the middle of the gel after overexpression of WT (1N4R), A152T (1N4R), P301S (1N4R) and WT (0N3R) tau using lentivirus particles were detected by Western blot of non-reducing SDS-PAGE using total tau antibody. Lentivirus particles were infected at MOI 3, and the cells were harvested on day 5 after tau overexpression. βIII tubulin antibody was used as a loading control. control: lentivirus without tau expression treatment group. (B) Immunofluorescence analysis of aggregated tau (oligomers and filaments) in hiPSC-derived neurons after tau overexpression using MC1 antibody (red). (−): lentivirus-untreated group. Neurons were treated with 1N4R tau lentivirus (MOI 10) and fixed on day 7 after tau overexpression. Scale bars, 100 µm. (C) Immunofluorescence analysis of tau oligomers in hiPSC-derived neurons after tau overexpression using T22 antibody (green). (−): lentivirus-untreated group. Neurons were treated with 1N4R tau lentivirus (MOI 10) and fixed on day 5 after tau overexpression. Even without exogenous tau introduction, we detected T22 signals in the cell somas. Representative tau overexpression-dependent signals are indicated by white bars and arrows. Scale bars, 100 µm.

To further confirm the contribution of tau overexpression for lowered cell viability, neurons were treated with siRNA against the tau-encoding *MAPT* gene (si-tau). Since tau overexpression induces cytotoxicity, we focused on the expression of exogenous 4R tau (Fig. 1D). In addition, we confirmed that si-tau also reduces the expression of endogenous 3R tau. Since endogenous 4R tau is not expressed in our culture system [26], the tau species present in this cellular model were considered to be endogenous 3R tau and overexpressed 4R tau (see also Fig. 2A). The exposure to si-tau suppressed the 4R tau expression induced by 1N4R LV infection (Fig. 1D), it also suppressed the endogenous GFP-tau expression without affecting neurite morphology (Supplementary Fig. S3A, B and S3E-H). The tau knockdown ameliorated the decreased neuronal viability by tau-overexpression compared to the control siRNA (Fig. 1B). In addition, the si-tau pretreatment protected neurons from injuries to the cell body and neurite caused by tau overexpression (Supplementary Fig. S3A-D). These results indicate that tau overexpression induces cell death in iPSC-derived neurons in a tau protein-dependent manner.

Furthermore, immunofluorescence analysis was performed to examine whether tau overexpression affects the levels of phosphorylated tau and other neuronal markers. By the infection of 1N4R tau containing lentivirus particles, the expression levels of 4R tau and phosphorylated tau (AT8, PHF1) were increased in neurons (Fig. 1E). The 4R tau expression was not detected in the neurons with the control lentivirus infection, indicating that the 4R tau signals in immunofluorescence imaging were derived from exogenous 1N4R tau overexpression. In addition, tau overexpression reduced neurites stained by βIII tubulin or MAP2 antibodies (Fig. 1E). These results suggest that tau overexpression either damaged neurites like we previously reported as an effect of a tau mutation [33].

The capability to aggregate is enhanced by increased expression of 4R tau or mutations such as P301S, while the A152T mutation induces neurotoxicity without enhancing the aggregation capability [34,35]. To compare the cytotoxic effects of different tau isoforms and mutations, lentiviruses for 1N4R, 0N3R (the shortest, fetal isoform), and mutant tau (A152T, P301S) overexpression were used to infect neurons. Cell viability was reduced in all tau overexpressing groups compared to the control group infected with lentivirus not overexpressing tau. On the other hand, the MOI-dependent decrease of viability did not significantly differ among different tau isoforms or mutations (Fig. 1F). These findings suggest that further enhancement of tau aggregation did not increase the toxicity.

### 3.2 Tau overexpression induces tau aggregation in iPSC-derived neurons

To determine whether tau was aggregated in neurons due to tau overexpression and to assess differences in aggregation among isoforms and mutations, non-reducing SDS-PAGE analysis was performed using lysates of neurons overexpressing WT (1N4R, the most abundant isoform), WT (0N3R, the shortest and fetus isoform), A152T (1N4R), or P301S (1N4R) tau proteins. Neurons treated with lentiviruses expressing WT (1N4R), A152T (1N4R), and P301S (1N4R) tau showed bands corresponding to 1N4R tau around 50 kDa, while neurons with the WT (0N3R) tau lentivirus showed bands corresponding to 0N3R tau. The GFP-tau band indicates endogenous expression, the 1N4R tau band by overexpression, and the 0N3R tau band by exogenous and endogenous expression. In addition, the signals of tau oligomer in the middle of the gel and larger aggregated tau at the top of the wells were observed in overexpression groups. The signals of tau oligomer and larger aggregated tau were speculated to correspond to tau aggregates (Fig. 2A). These results indicate that, regardless to the isoforms and mutations, tau formed aggregates in neurons through tau overexpression (Fig. 2A). These findings are consistent with the cytotoxicity data, indicating that the aggregation of exogenous tau has reached a plateau and the tau species with higher aggregation capacity did not further increase tau aggregation and toxicity.

Next, tau aggregation was confirmed by immunofluorescence analysis using MC1 (for tau conformational change and aggregation) [36] and T22 (for tau oligomers) [37] antibodies. MC1-positive (Fig. 2B) and T22-positive (Fig. 2C) neurite-like structures were observed in neurons overexpressing 1N4R tau, suggesting that tau aggregates, including tau oligomers, are likely mainly localized to neurites. The T22 immunofluorescent signals in the cell bodies were recognized in both 1N4R tau overexpression group and the lentivirus untreated (−) group, suggesting the signal in the cell body is likely non-specific staining of the T22 antibody. Since the cell body was not stained significantly in immunofluorescence analysis using the MC1 antibody, the specificity of the T22 antibody is assumed to be lower than that of the MC1 antibody. In addition, the T22 antibody has also been reported to show non-specific reactions in dot blot and flow cytometry [38]. The MC1 antibody is for academic use only, while the T22 antibody is the only commercially available tau oligomer antibody. We used the T22 antibody in the hope that this method could be used widely and continuously in future research. These findings also suggested the presence of tau aggregates in neurites of exogenous WT tau-expressing neurons.

### 3.3 The deletion of PHF6 domain ameliorates tau aggregation and cell death induced by tau overexpression

**Fig. 3:**
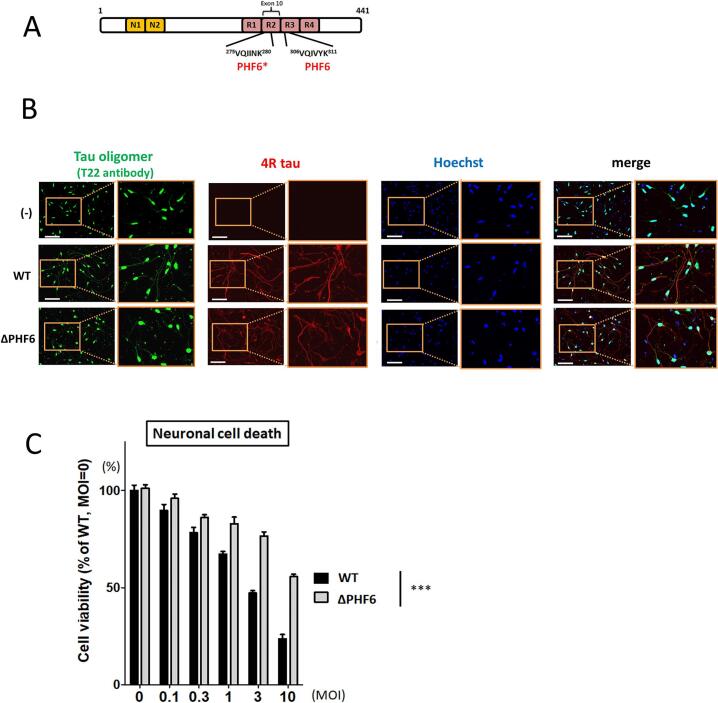
**PHF6 domain is involved in tau oligomer formation and cell death induced by tau overexpression.** (A) Schematic representation of PHF6* and PHF6 domains in tau protein. (B) Tau oligomer expression by WT and ΔPHF6 tau overexpression was detected by immunofluorescence using T22 tau oligomer antibody. 4R tau antibody was used as an expression control because 4R tau is expressed from exogenous tau but not from endogenous tau. Neurons were treated with lentivirus (MOI 10) and fixed on day 5 after tau overexpression. The PHF6 deletion reduced T22 signals but not RD4 signals in the neurites of the infected neurons. (−): lentivirus-untreated group. Scale bars, 100 µm. (C) Cell viability of iPSC-derived neurons at 5 days after WT or ΔPHF6 tau-overexpression (n = 3, mean + SD). ***: P ≤ 0.001 by analysis of covariance, WT vs. ΔPHF6.

To examine whether tau aggregation induces cell death in the tau-overexpressing neurons, we prepared lentiviral vector encoding tau lacking PHF6 (ΔPHF6) because the PHF6 domain within the tau molecule (Fig. 3A) is critical for tau aggregation [39]. First, to determine whether the PHF6 domain affects tau aggregation in iPSC-derived neurons, tau oligomerization was analyzed using T22 antibody in neurons overexpressing WT or ΔPHF6 tau. The 4R tau antibody could detect specifically exogenous tau because only 3R tau was expressed in our hiPSC-derived neurons as we previously described [26]. Therefore, 4R tau antibody was used to verify that not only WT but also ΔPHF6 1N4R tau are certainly expressed due to lentiviral infection. Therefore, it was reasonable that the obvious staining was not observed in the no treat (−) group. On the other hand, endogenous GFP-tau exhibits a neurite-like distribution, and the GFP-tau-positive neurites decreased according to the neurite damage and decrease of cell number caused by tau overexpression (Supplementary Fig. S2 and S3). Immunofluorescence analysis revealed that overexpression of ΔPHF6 tau resulted in decreased formation of tau oligomers compared to overexpression of WT tau, while 4R tau signals were detected at comparable level in both groups (Fig. 3B). Furthermore, we quantified the T-22 signals in the neurites, then found that ΔPHF6 1N4R tau displayed lower T22-positive neurite length than WT tau (Supplementary Fig. S4). T22 signals were increased mainly in neurites, therefore, this increase can be masked by mass detection procedures such as dot blotting. These results suggest that PHF6 contributes to the formation of tau oligomers in the neurites of this tau-overexpression model.

Next, in order to examine whether the lack of aggregation capability of ΔPHF6 affects the cytotoxicity of tau over-expression, we compared the cell viability of neurons overexpressing WT tau or ΔPHF6 tau. Neurons overexpressing ΔPHF6 tau showed reduced cytotoxicity compared to WT tau, indicating that ΔPHF6 tau is less toxic than WT tau (Fig. 3C). In addition, cell number counting also supported the reduced cytotoxicity of ΔPHF6 1N4R tau overexpression compared to WT 1N4R tau (Supplementary Fig. S2). These results suggest that the reduction of tau aggregation ameliorates cell death in this model.

### 3.4 Isoproterenol and epothilone D suppresses neuronal cell death due to tau overexpression

**Fig. 4:**
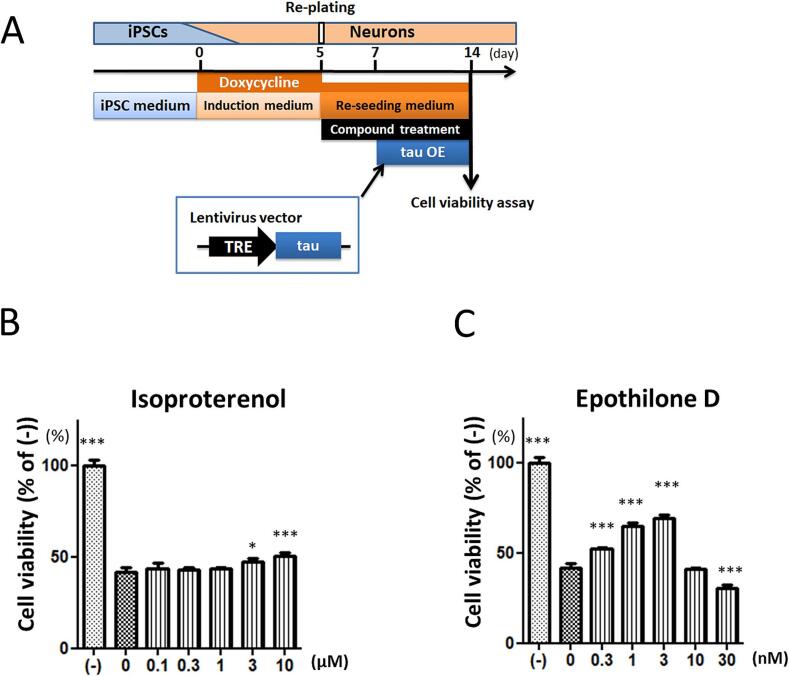
**Isoproterenol and epothilone D protected neurons against tau overexpression-induced cell death.** (A) Schematic diagram for evaluation of cell viability. Isoproterenol or epothilone D was added at the time of neuron plating. Neurons were treated with lentivirus (MOI 3) at 2 days after plating and cell viability was assessed 7 days after the lentivirus treatment. Neuronal viability of iPSC-derived neurons after exposure to tau aggregation inhibitor isoproterenol (B) and microtubule stabilizer epothilone D (C) were evaluated by CellTiter Glo assay (n = 3 ∼ 9, mean + SD). *: P ≤ 0.05, **: P ≤ 0.01, ***: P ≤ 0.001 by Dunnett’s test, 0 µM group vs. each group.

Given the confirmed effect of tau aggregation on cell death due to tau overexpression, we evaluated compounds to demonstrate the utility of this cellular model (Fig. 4A). First, we examined the effect of the tau aggregation inhibitor isoproterenol, which blocks tau oligomerization in vitro [40]. Isoproterenol increased the survival rate of hiPSC-derived neurons at high doses 3 and 10 µM (Fig. 4B). Next, we examined whether the indirect tau aggregation inhibitor, epothilone D (EpoD), could increase the viability in our assay system. EpoD is a microtubule stabilizer, and the binding of tau to the stabilized microtubules is thought to block tau-tau interaction and reduce tau aggregation, indirectly. EpoD decreased the viability at the highest dose of 10 nM, but increased viability at 0.3, 1, and 3 nM (Fig. 4C). In addition to the CellTiter Glo assay, we were also able to confirm the cytoprotective effects of isoproterenol and EpoD using the alamarBlue assay (Supplementary Fig. S5A, B). This reinforces the evidence that isoproterenol demonstrates weak but reproducible cytoprotective effects. Thus, our assay system can demonstrate the cytoprotective effects of a known tau aggregation inhibitor and a microtubule stabilizer, suggesting that this assay provides a platform for identifying novel neurodegenerative disease therapeutics.

## 4 Discussion

Our system offers a significant advantage for drug screening, requiring only a short two-week assay period by combining Ngn2 and miR-9/9*-124 expression, which accelerates neuronal differentiation from iPSCs. We validated this system by demonstrating the effects of established anti-tau compounds, thereby confirming its applicability as a drug discovery platform. While numerous cellular models of tau mutants exist, the majority of AD patients do not carry tau gene mutations, with the A152T tau variant being the sole known exception [26,41,42]. Therefore, cellular assay systems that replicate the toxicity of wild-type tau have garnered considerable attention. This study represents the first successful induction of tau-mediated neuronal death in human iPSC-derived neurons using wild-type tau, establishing a robust and efficient model for drug discovery targeting tauopathies and other neurodegenerative diseases.

In this study, we used an iPSC line with a heterozygous GFP-tau gene, in which the TagGFP2 gene was inserted into the 5′ end of the endogenous tau gene to express GFP-tau fusion protein based on the following advantages. This allows us to visualize the endogenous tau protein levels, subcellular localization in live cells. In this study, it was used to visualize the expression of endogenous tau (Supplementary Fig. S3). It is expected that further analysis of how the expression and distribution of tau change in live cells in response to extracellular stimuli will lead to a deeper understanding of neurodegenerative diseases.

In order to evaluate intracellular tau aggregation and screen tau aggregation inhibitors in cell models, cell lines such as HEK293, Neuro2a, and SH-SY5Y were used so far [24,43,44]. Furthermore, for induction of cell death, aggregation enhancers were required in addition to tau overexpression. For instance, in HEK293 cells, the concomitant use of tau overexpression and an aggregation enhancer, Congo red, induced cell death [24]. Tau overexpression using AAV vectors and treatment of tau aggregation seeds in 3D culture of iPSC-derived neural progenitor cells induces tau aggregation and cell death [25]. Additionally, tau aggregates are formed by adding the four-repeat domain (K18) fragment of tau to neuronal cells or introducing mutant PS1 and APP into 3D culture [45,46,47]. Thus, complex systems involving long culture periods, combinations of compound treatments, or over-expression of double mutant transgenes have been used to induce cellular tau aggregation in neuronal cell models. In contrast to these models, we established a simple human cellular model that displays wild-type tau aggregation-dependent neuronal cell death in a short period. Our method mainly differs in the following three points. 1) Overexpression of exogenous wild-type tau using a high-expression promoter, the tetO promoter, promoted the formation of toxic tau aggregates even in the absence of pre-aggregated recombinant tau seeds. 2) Since one of the neurotoxicity mechanisms due to tau is mediated by hyperexcitability in glutamatergic neurons, we employed a rapid induction of functionally active neurons by a method using Ngn2 and miR-9/9*-124 expression [29]. 3) Compared with other transfection methods, infection efficiency, gene transfer, or expression level into neurons by lentivirus may be highly efficient.

In addition to the decreased cell viability, tau overexpression also affected neuronal processes, which was revealed by the reduction of βⅢ-tubulin and MAP2-positive neurites. In this cellular model, exogenous tau was induced to the pre-differentiated neurons to avoid the possibility that tau overexpression inhibits neurite outgrowth and neuronal maturation. However, it is still possible that that hTau expression inhibited additional neurite outgrowth and neuronal maturation. To exclude the possibility, we also examined the hTau LV infection to hiPSC-derived neurons after one week of differentiation. However, since the infection efficiency of lentivirus to hiPSC-derived neurons was decreased significantly after one week of differentiation, it was not possible to estimate the degree of contribution of cell death and inhibition of neuronal maturation after one week of differentiation.

The hexapeptide domains, PHF6 and PHF6* domains, in the microtubule-binding repeats of tau have a high propensity to form β-sheet structures and constitute the core of tau filaments [48,49,50]. Actually, in our experiments, deletion of the PHF6 domain reduced tau aggregation, and we also observed a corresponding reduction in neuronal cell death. Interestingly, no significant difference was observed in tau aggregation and cell death between 3R tau (containing only PHF6) and 4R tau (containing PHF6 and PHF6*) overexpression. A previous study reported PHF6 but not PHF6* peptides aggregation in cells [51]. Tau dimerization in cells was more drastically reduced by the deletion of PHF6 than the deletion of PHF6*, suggesting that the PHF6 domain is more potent in aggregation than the PHF6* domain [39]. These findings indicate the higher aggregation propensity of PHF6 over PHF6* and can explain why tau aggregation did not differ between 3R tau and 4R tau, while PHF6 deletion reduced tau aggregation. Thus, our findings that the PHF6 peptide is critical for tau aggregation are consistent with previous studies.

It is possible that tau aggregation-independent cell death mechanisms are present in this cellular model. Although the appearance of T22 antibody-positive neurites was sufficiently suppressed by PHF6 deletion, ΔPHF6 tau overexpression also caused MOI-dependent cytotoxicity. Tau protein that is not bound to microtubules shows toxicity mediated by calcium influx [52]. Taken together, tau overexpression may induce both of tau aggregation dependent and independent toxicities in our assay system.

Although we initially predicted that the P301S mutation would enhance tau aggregation and cell death, such enhancement was not observed. It was suggested that the tau aggregation by WT tau overexpression tau may be sufficiently intense to mask the effects of the mutation. P301S tau has less capability to bind to microtubules and more propensity to aggregate than WT [51,53]. However, gene editing from WT to P301S of endogenous tau was not enough to trigger neuronal cell death and tau aggregation [26], indicating that the P301S mutation alone is not sufficient to induce tau aggregation and neuronal cell death. Thus, the high level of exogenous tau expression driven by the tetO promoter was more potent to induce aggregation than the P301S mutation.

In this study, it remains to be determined which tau aggregate species are linked to neurotoxicity and by which mechanisms tau aggregates induce neuronal cell death. We detected tau oligomers but did not discriminate them from filaments, and the deletion of PHF6 peptides reduced aggregates, including oligomers and filaments [39]. Thus, further investigation is required to determine which of these forms are toxic to neurons. Additionally, we did not explore various intracellular mechanisms such as protein homeostasis pathways of autophagy, lysosomes, proteasomes, and heat shock proteins, which might be involved in the cytotoxicity induced by aggregated tau [54]. These pathways are involved in impairment caused by other proteins associated with other neurodegenerative diseases. For example, impairment of protein homeostasis pathways is recognized in neurodegeneration cellular models overexpressing mutant α-synuclein [55] and mutant γPKC [56,57]. Therefore, additional mechanistic analysis of tau-dependent neuronal cell death in this model will help to understand the mechanisms of neuronal loss in neurodegenerative diseases.

## Data availability

The data that support the findings of this study are available from the corresponding author upon request.

## CRediT authorship contribution statement

**Hirokazu Tanabe:** Writing – original draft, Visualization, Methodology, Investigation. **Sumihiro Maeda:** Writing – original draft, Supervision, Project administration, Methodology, Investigation, Funding acquisition, Conceptualization. **Etsuko Sano:** Resources. **Norio Sakai:** Writing – original draft. **Setsu Endoh-Yamagami:** Writing – original draft, Supervision, Project administration, Conceptualization. **Hideyuki Okano:** Writing – review & editing, Supervision, Project administration, Funding acquisition, Conceptualization.

## Declaration of competing interest

Hirokazu Tanabe and Setsu Endoh-Yamagami are employees of Fujifilm Corporation. Hideyuki Okano received a research grant from Fujifilm Corporation. Hirokazu Tanabe, Sumihiro Maeda, Setsu Endoh-Yamagami, and Hideyuki Okano are inventors on a patent application (JP2022-157516, US2022/0315890) for “Nerve cell and application thereof”. All other authors have no competing interests.
