# Supplementary material for: Tau aggregation induces cell death in iPSC-derived neurons
Source: Aging Brain. 2025 Apr 11;7:100136. doi: 10.1016/j.nbas.2025.100136 (PMC12018045; doi:10.1016/j.nbas.2025.100136)
Supplement: Supplementary Data 1 [file mmc1.docx]

**Tau aggregation induces cell death in iPSC-derived neurons**

**Hirokazu Tanabe^a^, Sumihiro Maeda*^b^, Etsuko Sano^c^, Norio Sakai^d^, Setsu Endoh-Yamagami*^a^, and Hideyuki Okano*^b, c^**

^a^ FUJIFILM Corporation, Bio Science & Engineering Laboratories, Kanagawa, Japan

^b^ Department of Physiology, Keio University School of Medicine, Tokyo, Japan

^c^ Keio University Regenerative Medicine Research Center, Kanagawa, Japan

^d^ Department of Molecular and Pharmacological Neuroscience, Graduate School of Biomedical & Health Sciences Hiroshima University, Hiroshima, Japan.

*Corresponding authors:

Sumihiro Maeda, Department of Physiology, Keio University School of Medicine, 35 Shinanomachi, Shinjuku-ku, Tokyo, Japan, e-mail: [sumihiro.maeda@keio.jp](mailto:sumihiro.maeda@keio.jp),

Setsu Endoh-Yamagami, FUJIFILM Corporation, Bio Science & Engineering Laboratories, 577 Ushijima, Kaisei-machi, Ashigarakami-gun, Kanagawa, e-mail: setsu.endo@fujifilm.com

Hideyuki Okano, Keio University Regenerative Medicine Research Center, 3-25-10 Tonomachi, Kawasaki-ku, Kawasaki, Kanagawa, Japan, e-mail: [hidokano@keio.jp](mailto:hidokano@keio.jp) (lead contact)

**Supplementary Figure**

**Supplementary Fig. S1**: **Tau overexpression induces cell death in iPSC-derived neurons.**

Cell viability of iPSC-derived neurons at 4 and 6 days after 1N4R tau overexpression was evaluated by alamarBlue assay. The neurons were prepared according to the method in Supplementary Method S1, and the alamarBlue assay was performed according to the Supplementary Method in S2. The survival rate of neurons decreases with the length of days in culture (mean+SD). ****: P ≤ 0.0001 by one-sample t-test against 1.

**Supplementary Fig. S2**: **Tau aggregation due to tau overexpression decreased the cell number.**

Neurons were prepared according to the method 2.3., and tau overexpression was performed according to the method 2.4.. Neurons were treated with WT of ΔPHF6 1N4R lentivirus (MOI 10) and fixed on day 5 after tau overexpression. Neurons were stained by Hoechst 33342 and counted the number of cells (mean+SD). **: P ≤ 0.01, ***: P ≤ 0.001 by Tukey’s multiple comparison test. (-): lentivirus-untreated group.


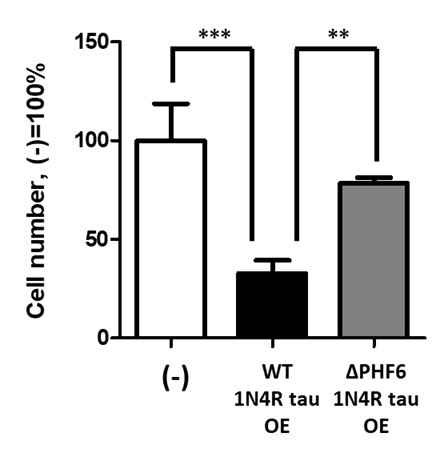


**Supplementary Fig. S3***:* **Observation of endogenous tau expression and neurite morphology in living neurons.**

si-tau treatment and tau overexpression were performed on neurons according to the scheme shown in Fig. 1. Since the neurons express endogenous GFP-tau, phase contrast (A-D) and GFP fluorescence (E-H) images were obtained from live cells without fixation. Scale bars, 100 µm

**Supplementary Fig. S4: Tau overexpression induces tau aggregation, and PHF6 domain is involved in tau oligomer formation.**

The length of T22-positive neurites was measured as an indicator of formation of tau oligomers in neurites according to Supplementary Method S3. (n=3, mean+SD). *: P ≤ 0.05, ns (not significant): P > 0.05 by Tukey’s multiple comparison test. (-): lentivirus-untreated group.


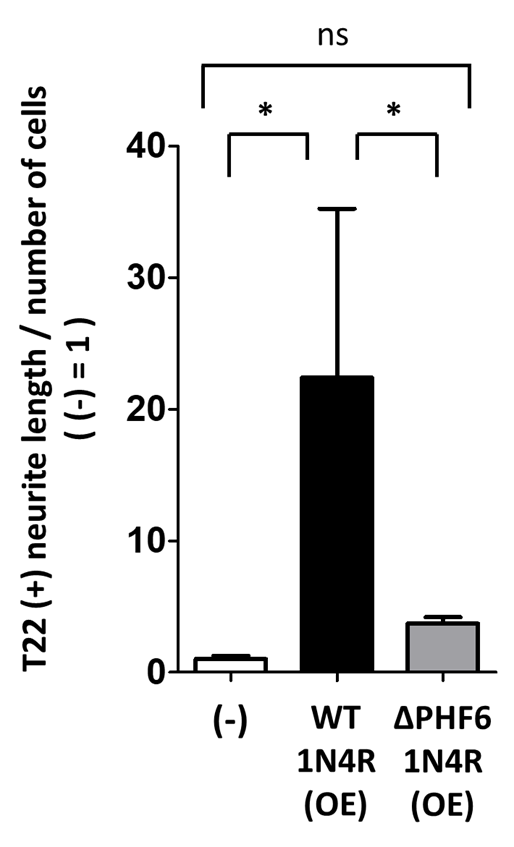


**Supplementary Fig. S5: Isoproterenol and epothilone D protected neurons against tau overexpression-induced cell death.**

Cell viability of neurons overexpressing tau treated with isoproterenol (A) or EpoD (B) was evaluated by alamarBlue assay. The neurons were prepared according to the method in Supplementary Method S1, and the alamarBlue assay was performed according to the Supplementary Method in S2 (n=8, mean+SD). ****: P ≤ 0.0001, ***: P ≤ 0.001, *: P ≤ 0.05, ns (not significant): P > 0.05 by Dunnett’s multiple comparisons test against vehicle control with LV infection (DMSO).

A　　　　　　　　　　　　　　　B

**Supplementary Method**

***Supplementary Method S1. Preparation of neurons***

Human iPSCs were dissociated using 0.5×TrypLE Select, and 2.5x10^4^ cells (96 well-plate) were seeded with neuronal induction medium (Neurobasal Plus medium containing 2% B27 Plus supplement, 1% CultureOne supplement, 1% GlutaMAX, 200 µM L-ascorbic acid, 200 µM dbcAMP, 10 µM Y27632, 20 µM DAPT and 4 µg/ml doxycycline (DOX)) onto 96-well culture plates pre-coated with 0.0001% poly-L-lysine and iMatrix-511 silk. On days 5, whole medium was changed with fresh neuronal maintenance medium (Neurobasal Plus medium containing 2% B27 Plus supplement, 1% CultureOne supplement, 1% GlutaMAX, 200 µM L-ascorbic acid, 200 µM dbcAMP, and 10 ng/ml BDNF). Half of the medium was exchanged with the fresh medium every 4–7 days after day 6.

***Supplementary Method S2. AlamarBlue assay***

To quantify cell viability, the maintenance medium was exchanged with fresh medium containing sodium resazurin (FUJIFILM Wako Pure Chemical) at 100μM, and the cells were incubated for 4-6 hours at 37ºC. Then, the fluorescence of resazurin was measured with a multimode microplate reader EnSpire (PerkinElmer, Japan) at excitation 560 nm/emission 590 nm.

***Supplementary Method S.3. Measurement of T22-positive neurite length for an indicator of tau aggregation***

The length of T22-positive neurites was measured as an indicator of formation of tau oligomers in neurites. The total length of T22 neurites in each image was calculated using Image J. The total length of T22 neurites in each image was then normalized to the number of cells in each image. To exclude dead cells, the number of cells was counted in the overlapping area of Hoechst 33342-positive and T22-positive cell body-like structures.
